# HBV and HCV seroprevalence and their correlation with CD4 cells and liver enzymes among HIV positive individuals at University of Gondar Teaching Hospital, Northwest Ethiopia

**DOI:** 10.1186/1743-422X-10-171

**Published:** 2013-05-30

**Authors:** Yitayih Wondimeneh, Meseret Alem, Fanaye Asfaw, Yeshambel Belyhun

**Affiliations:** 1 School of Biomedical and Laboratory Sciences, College of Medicine and Health Sciences, University of Gondar, P.O. Box 196, Gondar, Ethiopia; 2 Institute of Virology, Faculty of Medicine, University of Leipzig, Johannisallee 30, Leipzig, 04103, Germany

**Keywords:** HBV, HCV, HIV, CD4 T cells, Liver enzymes

## Abstract

**Background:**

The co-existence of viral hepatitis caused by HBV and HCV become common causes of severe liver complication and immunological impairment among HIV infected individuals. The aim of this study was to assess the seroprevalence of HBV and HCV and their correlation with CD4 and liver enzyme levels among HAART naïve HIV positive individuals.

**Method:**

A Cross-sectional study was conducted from March-May, 2011 at University of Gondar Teaching Hospital, Northwest Ethiopia. HBV and HCV serological tests and liver enzymes as well as CD4 T cell level determination were assessed following the standard procedures. Socio-demographic data was collected by using structured questionnaire. The data was entered and analyzed by using SPSS version 20.0 statistical software and p < 0.05 was considered as statistically significant.

**Result:**

Among 400 study participants, the overall prevalence of HIV-viral hepatitis co-infection was 42(11.7%). The prevalence of HIV-HBV, HIV-HCV and HIV-HBV-HCV co-infections were 20(5.6%), 18(5.0%) and 4(1.1%) respectively. Study participants who had HIV-HBV, HIV-HCV and HIV-HBV-HCV co-infection have relatively raised mean liver enzyme levels (ALT, AST and ALP) than HIV mono-infected once. Individuals with HIV-HBV, HIV-HCV and HIV-HBV-HCV co-infection also had a lower mean CD4 levels than HIV mono-infected study participants. The mean CD4 value in males was lower than females.

**Conclusion:**

The prevalence of HBV and HCV was higher than reports from general population of the country. Raised levels of liver enzymes and lowered mean CD4 counts were seen in HIV-HBV, HIV-HCV and HIV-HBV-HCV co-infections. These findings underscore the importance of screening all HIV positive individuals before initiating antiretroviral treatment.

## Introduction

Hepatitis B Virus (HBV) and Hepatitis C Virus (HCV) are the most common cause of chronic liver diseases worldwide. The infection continues to have a severe and invasive impact on the health of millions of people around the world and the infection is often asymptomatic [1-4]. Due to similar routes of viral transmission [5], co-infection of HIV with HBV and/or HCV is common. The co-infection pattern of these viruses showed that 10.0% of the HIV-infected population estimated to have chronic HBV infection and around a third estimated to have chronic HCV infection worldwide [2,5]. However, studies reported [6-9] that the rates of co-infection of HIV with either HCV or HBV vary from region to region, study population and risk factors for HIV acquisition. A systematic review in 18 sub Saharan African countries also showed that the prevalence of HBV and HCV in HIV-infected individuals ranged from 3.9-7.3% and 6.9%, respectively [10].

Despite the effective decline of the mortality and morbidity rate from HIV/AIDS as the result of highly active antiretroviral therapy (HAART), liver diseases due to chronic HBV and HCV infections become a leading cause of death. Although the direct impact of HCV upon HIV disease progression remains controversial in many reports [8,11-15], the complex interactions between HIV-HBV/HCV co-infection and HAART are increasingly apparent in HIV disease progression [16].

In HIV-HBV co-infections, HIV infection causes increased rates of persistent HBV infection, increased cirrhosis and liver-related mortality and increased risk of hepatocellular carcinoma at lower CD4T cell counts [17]. Similarly in HIV-HCV co-infections, there is a more rapid progress to cirrhosis, end-stage liver disease and hepatocellular carcinoma [18].

The impact of HBV and HCV could not limited in causing liver hepatotoxicity but also results in failure in immunological recovery in HIV positive patients. For example, a study in Tanzania reported slow rate of immunologic recovery after initiation of HAART treatment and higher risk of hepatotoxicity among HIV/HBV and HIV/HCV co-infected patients [19]. Thus the management of HBV and HCV in HIV infection is complicated and bring high burden in particular where HIV is rampant. As the result, globally HIV, HBV and HCV become the major public health concerns [20,21]. In some countries, screening of HIV-infected individuals for HBV and HCV is highly recommended before initiation of antiviral treatment [22].

In Ethiopia, the seroprevalence of HBV and HCV among HIV positive individuals is scarce except few reports among blood donors and infection prone groups [23-25]. In addition, there is no report about liver enzyme levels and CD4 count determination among HIV-HBV and/or HIV-HCV co-infected patients. Therefore, the aim of this study was to assess the seroprevalence of HBV and HCV and CD4 cells as well as liver enzyme levels among HIV positive individuals at University of Gondar Teaching Hospital, Northwest Ethiopia.

## Materials and methods

### Study design, area, and period

A cross-sectional study was conducted from March to May, 2011 at University of Gondar Teaching Hospital, which is found in Gondar town, Northwest Ethiopia.

### Source population and study participants

The source populations were all HIV positive individuals who had access to be served at University of Gondar Teaching Hospital. The study participants were all HAART naïve HIV positive adult individuals who had visited ART clinics at Gondar University Hospital during the study period. A total of 403 study participants were enrolled by considering 95% confidence interval, 5% margin of error, 50% proportion (since there is no previous estimation of HBV and HCV among HIV infected individuals in the area) and 5% contingency. However, 2(0.5%) of them had refused to participate and the other 1(0.2%) were excluded due to chronic alcoholism and 400(99.3%) HIV positive individuals were used for the final analysis.

### Inclusion and exclusion criteria

All ART naïve adult HIV positive individuals who visited ART clinic for CD4 and liver enzyme level determinations for their pre-ART follow up during the study period were included. But individuals who were on ART follow up and those ART naïve but who refused to give informed consent were excluded from the study. Individuals who have been vaccinated against HBV and those who visited ART clinic and requested for laboratory investigation for the second and consequent follow up at the time of the study period were also excluded from the study to avoid duplication. Patients who had TB, malaria, leishmaniasis, opportunistic infections (OIs), drug induced hepatotoxicity and chronic alcoholism were assessed and excluded as per the HIV management guidelines of Ethiopia [26].

## Data collection procedures

Socio-demographic information and other relevant possible risk factors of the study participants were collected using structured and pre tested questionnaire by trained nurses and physicians. Ten milliliter (10 ml) of veinous blood was aseptically collected using plain and EDTA vacutainer tube (5 ml in each tube) for the determination of HBV and HCV seroprevalence and CD4 and liver enzyme levels from each study participants. The blood specimen in the plain tube was centrifuged at 3000 RPM for 5 minutes to separate the serum and used for determination of liver enzyme levels within one hour of separation. The remaining serum kept in deep refrigerator (−40°C) until detection of HBV and HCV. The second tube that contains whole blood was used for the CD4 levels determination.

The collected sera were checked for the presence of HBsAg using TULIP’S INSTANT (TULIP DIAGNOSTICS (P) LTD. 88/89, Phase II C, Verna Ind. Est., Verna, Goa-403 722, INDIA) which have sensitivity and specificity of 100%. Anti-HCV antibody was detected using Flavicheck-HCV WB (TULIP DIAGNOSTICS (P) LTD. 88/89, Phase II C, Verna Ind. Est., Verna, Goa-403 722, INDIA) following the manufacturer’s instructions. The catalytic activities of the liver enzyme levels were analyzed by using clinical chemistry analyzer (Humastar 80) and the CD4 count was done by BD FACS count flow cytometry machine.

### Quality control

The standard operational procedures were strictly followed for the quality control issues. Both hepatitis B and C kits were checked by using known HBsAg and anti-HCV antibody positive and negative control samples. Similarly the quality of both CD4 and liver enzyme reagents were regularly monitored by running control materials in each morning before the actual work was done.

## Data analysis

The data was entered and analyzed using SPSS Version 20.0 statistical software and the differences in proportions was evaluated by Pearson’s Chi-square test (x^2^ test) and P-value of less than 0.05 was considered as statistical significant. Mean plus standard deviation with 95% confidence interval (CI) was also used for continuous variables and the difference in means was compared with independent-sample t-test.

### Ethical consideration

The study was conducted after obtaining ethical clearance from ethical committee of Department of Medical Microbiology, Immunology and Parasitology, University of Gondar (University of Gondar, College of Medicine and Health Sciences, Ref. No: V/D06/07/2011). Informed consent was also obtained from each study participants.

## Result

### Socio-demographic characteristics

Among 400 study participants, 122(30.5%) were males (mean age: 37 ± 9 years) and 278(69.5%) were females (mean age: 32 ±9 years) with male to female ratio of 0.4:1. The lowest and the highest age of the study participants were 18 and 70 years respectively. The median age of the study participants was 32 years. Majority 167 (41.8%) of the study participants were in the age group of 30–39 years old. Among the study groups, 151 (37.7%) were illiterate (Table 1).

**Table 1:** Socio-demographic characteristics of HIV positive study participants at University of Gondar Teaching Hospital, Northwest Ethiopia, 2011

**Characteristics**	**Male N (%)**	**Female N (%)**	**Total N (%)**
**Age group**			
18-29	21(16.4)	107(83.6)	128(32.0)
30-39	50(29.9)	117(70.1)	167(41.8)
40-49	40(51.3)	38(48.7)	78(19.5)
≥50	11(40.7)	16(59.3)	27(6.8)
**Marital status**			
Single	27(36.0)	48(64.0)	75(18.8)
Married	76(38.0)	124(62.0)	200(50.0)
Divorced	17(20.7)	65(79.3)	82(20.5)
Widowed	2(4.7)	41(95.3)	43(10.7)
**Religion**			
Christian	111(30.2)	256(69.8)	367(91.8)
Muslim	11(33.3)	22(66.7)	33(8.2)
**Education**			
Illiterate	34(22.5)	117(77.5)	151(37.7)
Elementary	31(32.3)	65(67.7)	96(24.0)
High school	37(33.3)	74(66.7)	111(27.8)
Certificate and above	20(47.6)	22(52.4)	42(10.5)
**Residence**			
Urban	103(30.7)	232(69.3)	335(83.7)
Rural	19(29.2)	46(70.8)	65(16.3)
**Occupation**			
Civil servant	33(56.9)	25(43.1)	58(14.5)
Merchant	18(42.9)	24(57.1)	42(10.5)
Daily laborer	21(52.5)	39(97.5)	60(10.0)
Farmer	15(60.0)	10(40.0)	25(6.3)
House wife		114(100)	114(28.5)
Student	3(42.9)	4(57.1)	7(1.8)
Driver	8(80.0)	2(20.0)	10(2.5)
Commercial sex worker		5(100.0)	5(1.3)
No work	7(14.6)	41(85.4)	48(12.0)
Other	17(54.8)	16(51.6)	31(7.7)

### Seroprevalence of HBVand HCV

The overall prevalence of viral hepatitis (HBV and HCV) was 42(11.7%). The seroprevalence of HBV and HCV were 20(5.6%) and 18(5.0%), respectively. Only 4(1.1%) of HIV positive individuals showed triple (HIV-HBV-HCV) infections.

### HIV-HBV and HIV-HCV co-infection and sociodemographic characteristics

Significantly higher prevalence of HIV-HBV co-infection was observed in males 11(9.4%) compared to females 9(3.4%) (*X*^2^ = 5.714, P = 0.017). Although statistically non-significant, higher 6(8.3%) (*X*^2^ = 3.083, P = 0.379) prevalence of HIV-HBV co-infection was observed in the age group between 40–49 years. Individuals who were widowed 4(9.8%) (*X*^2^ =3.681, P = 0.298) and those who had better educational status (certificate, diploma and above) 4(10.3%) (*X*^2^ = 2.602, P = 0.457) showed non-significantly higher HIV-HBV positive rate. The prevalence of HIV-HBV co-infection in urban and rural residences were 17(5.3%) and 3 (5.0%) respectively. Non-statistically significant higher prevalence of HIV-HCV co-infection was also observed in females 15(5.6%), in the age group of 40–49 years 5(7.0%), in rural residence 4(6.6%), in married 12(6.3%) and in the housewives 8(7.2%). The co-existence of both HBV and HCV in males and females were 2(1.8%) and 2(0.8%) (*X*^2^ = 0.786, P = 0.375) respectively (Table 2).

**Table 2:** Sociodemographic characteristics and their association with HIV-HBV and HIV-HCV co-infections at University of Gondar Teaching Hospital, Northwest Ethiopia, 2011

**Variables**	**HIV-HBV**			**HIV- HCV**			**HIV-HBV-HCV**		
	**Negative N (%)**	**Positive N (%)**	**Sign**	**Negative N (%)**	**Positive N (%)**	**Sign**	**Negative N (%)**	**Positive N (%)**	**Sign**
**Age**									
18-29	118(95.9)	5(4.1)	*X*^2^ = 3.083	118(96.7)	4(3.3)	*X*^2^ = 1.461	118(99.2)	1(0.8)	*X*^2^ = 12.169
30-39	150(94.3)	9(5.7)	P = 0.379	150(94.9)	8(5.1)	P = 0.691	150(100.0)	0(0.0)	P = 0.007
40-49	66(91.7)	6(8.3)		66(93.0)	5(7.0)		66(98.5)	1(1.5)	
≥50	24(100)	0(0)		24(96.0)	1(4.0)		24(92.3)	2(7.7)	
**Sex**									
Male	106 (90.6)	11(9.4)	*X*^2^ = 5.714	106(97.2)	3(2.8)	*X*^2^ = 1.395	106(98.2)	2(1.8)	*X*^2^ = 0.786
Female	252(96.6)	9(3.4)	P = 0.017	252(94.4)	15(5.6)	P = 0.238	252(99.2)	2(0.8)	P = 0.375
**Residence**									
Urban	301(94.7)	17(5.3)	*X*^2^ = 0.12	301(95.6)	14(4.4)	*X*^2^ = 0.501	301(99.0)	3(1.0)	*X*^2^ = 0.242
Rural	57(95.0)	3(5.0)	P = 0.913	57(93.4)	4(6.6)	P = 0.479	57(98.3)	1(1.7)	P = 0.623
**Marital status**									
Single	72(96.0)	3(4.0)	*X*^2^ = 3.681	72(100.0)	0(0.0)	*X*^2^ = 4.653	72(100.0)	0(0.0)	*X*^2^ = 1.777
Married	178(96.2)	7(3.8)	P = 0.298	178(93.7)	12(6.3)	P = 0.199	178(98.3)	3(1.7)	P = 0.620
Divorced	71(92.2)	6(7.8)		71(94.7)	4(5.3)		71(98.6)	1(1.4)	
Widowed	37(90.2)	4(9.8)		37(94.9)	2(5.1)		37(100.0)	0(0.0)	
**Religion**									
Christian	327(94.5)	19(5.5)	*X*^2^ = 0.327	327(94.8)	18(5.2)	*X*^2^ = 1.699	327(99.1)	3(0.9)	*X*^2^ = 1.311
Muslim	31(96.9)	1(3.1)	P = 0.567	31(100.0)	0(0.0)	P = 0.192	31(96.9)	1(3.1)	P = 0.252
**Education**									
Illiterate	133(94.3)	8(5.7)	*X*^2^ = 2.602	133(95.0)	7(5.0)	*X*^2^ = 0.672	133(97.8)	3(2.2)	*X*^2^ = 4.553
Elementary	89(95.7)	4(4.3)	P = 0.457	89(96.7)	3(3.3)	P = 0.880	89(100.0)	0(0.0)	P = 0.208
High school	101(96.2)	4(3.8)		101(94.4)	6(5.6)		101(100.0)	0(0.0)	
Certificate & above	35(89.7)	4(10.3)		35(94.6)	2(5.4)		35(97.2)	1(2.8)	
**Occupation**									
Civil servant	49(89.1)	6(10.9)	*X*^2^ = 12.352	49(96.1)	2(3.9)	*X*^2^ = 4.427	49(98.0)	1(2.0)	*X*^2^ = 5.695
Merchant	37(90.2)	4(9.8)	P = 0.194	37(97.4)	1(2.6)	P = 0.881	37(100.0)	0(0.0)	P = 0.770
Daily laborer	54(91.5)	5(8.5)		54(98.2)	1(1.8)		54(100.0)	0(0.0)	
Farmer	22(95.7)	1(4.3)		22(95.7)	1(4.3)		22(95.7)	1(4.3)	
House wife	103(99.0)	1(1.0)		103(92.8)	8(7.2)		103(99.00)	1(1.0)	
Student	7(100.0)	0(0.0)		7(100.0)	0(0.0)		7(100.0)	0(0.0)	
Driver	9(90.0)	1(10.0)		9(100.0)	0(0.0)		9(100.0)	0(0.0)	
Com. sex worker	4(100.0)	0(0.0)		4(100.0)	0(0.0)		4(100.0)	0(0.0)	
No work	44(97.8)	1(2.2)		44(93.6)	3(6.4)		44(100.0)	0(0.0)	
Other	29(96.7)	1(3.3)		29(93.5)	2(6.5)		22(95.7)	1(4.3)	

### Liver enzyme levels and mean CD4 count in HIV/HBV, HIV/HCV and HBV/HCV/HIV co-infection

The mean serum levels; ALT, AST, and ALP in HIV mono-infected study participants were 25 international units (IU), 27 IU and 243 IU respectively. However, in HIV-HBV co-infected study participants, the levels of ALT, AST and ALP were non-significantly raised (29 IU, 31 IU and 262 IU, respectively). Similarly, in HIV-HCV co-infected study participants, the mean levels of ALT, AST and ALP were 27 IU, 32 IU, and 290 IU respectively. Statistically non-significant raised mean serum ALT, AST and ALP were found in HIV-HBV-HCV triple infected study participants (Table 3).

**Table 3:** Mean CD4 and liver enzyme levels and their association with HBV and HCV co-infection at University of Gondar Teaching Hospital, Northwest Ethiopia, 2011

**Immunological and liver biomarkers**	**HIV alone**	**HIV/HBV**	**P-value**	**HIV alone**	**HIV/HCV**	**P-value**	**HIV alone**	**HIV/HBV/HCV**	**P-value**
**CD4 mean ± SD**	288 ± 190	250	0.375	288 ± 190	274 ± 138	0.754	288 ± 190	125 ± 96	0.087
Normal value	500-1300	500-1300		500-1300	500-1300		500-1300	500-1300	
**ALT mean ± SD**	25 ± 21	29 ± 18	0.356	25 ± 21	27 ± 17	0.472	25 ± 21	39 ± 6	0.170
Normal value	0-37	0-37		0-37	0-37		0-37	0-37	
Abnormal High (%)	12.3%	20%		12.3%	22.2%		12.3%	50%	
**AST mean ± SD**	27 ± 19	31 ± 18	0.419	27 ± 19	32 ± 17	0.339	27 ± 19	33 ± 15	0.587
Normal value	0-34	0-34		0-34	0-34		0-34	0-34	
Abnormal High (%)	20.1%	25%		20.1%	27.8%		20.1%	50%	
**ALP mean ± SD**	243 ± 130	262 ± 118	0.515	243 ± 130	290 ± 127	0.135	243 ± 130	332 ± 228	0.176
Normal value	72-306	72-306		72-306	72-306		72-306	72-306	
Abnormal High (%)	19.6%	15%		19.6%	33.3%		19.6%	50%	

The mean CD4 count of HIV mono-infection was 288cells/mm^3^. However, in HIV-HBV, HIV-HCV and HIV-HBV-HCV co-infections, the mean CD4 count were 250 cells/mm^3^, 274 cells/mm^3^ and 125 cells/mm^3^ respectively. However, the difference was not statistically significant (Table 3). The CD4 value in females was higher than males (299 ± 197 vs 249 ± 152). Males study participants who had both HBV and HCV have the lowest mean CD4 count. The highest and lowest mean CD4 values were observed in the age groups of 18–29 and ≥50 years respectively (Table 4).

**Table 4:** Mean CD4 values in relation to gender and different age categories at University of Gondar Teaching Hospital, Northwest Ethiopia, 2011

**Variables**	**Mean CD4 count**				
	**HIV alone**	**HIV-HBV**	**HIV-HCV**	**HIV-HBV-HCV**	**Over all**
**Gender**					
Male	256 ±155	197 ± 113	334 ± 133	75 ± 30	249 ± 152
Female	302 ± 201	314 ± 188	262 ± 140	176 ± 129	299 ± 197
**Age categories**					
18-29	338 ± 223	383 ± 201	328 + 151	84 ± 0	337 ± 219
30-39	261 ± 151	169 ± 108	241 + 128		255 ± 149
40-49	292 ± 203	260 ± 121	286 + 173	53 ± 0	286 ± 196
≥50	203 ± 135		260 +0	182 ± 121	204 ± 129

## Discussion

This study investigated the seroprevalence of HBV and HCV among HIV positive study participants and tried to assess levels of liver enzymes and CD4 count for HIV mono infected, HIV-HBV and HIV-HCV co-infected and HIV-HBV-HCV triple infected individuals. The overall prevalence (11.7%) of hepatitis (both HBV and HCV) among the study participants was very high. In this study, HIV-HBV co-infection rate was 5.6% which is more or less comparable with 7.1% prevalence [24] among blood donors in the same hospital. However, the present prevalence was lower as compared to studies reported in Nigeria (9.2%) [27], Ethiopia (10.9%) [23] and Malawi (20.4%) [28]. In the present study, the prevalence of HIV-HBV co-infection was higher in males than females (9.4%% vs 3.4%) which are in line with some other reports [29-31]. Generally, as several studies reported and anticipated in different parts of the world, such co-infection differences could be due to differences in geographic regions, types of risk groups and the means of exposures involved [26,27,32-35].

The seroprevalence rates of HIV-HCV co-infection in this study was 5.0% which is almost comparable with the studies which were done in Nigeria (5.8%), Malawi (5.0%), Burkina Faso (4.8%) and Senegal (8.0%) [27,28,36,37]. However, the epidemiological survey of HCV in Ethiopia showed variation from 2-3% in the general population in early 1990s [38-40] and recently, the co-infection rates of HIV-HCV ranges from 3.6-13.3% in different reports [24,25,41-45]. The reasons for the HCV variation both in HIV infected individuals and the general population could share the factors responsible HBV prevalence variations discussed above.

The seroprevalence of HIV-HBV-HCV triple infection in this study was 1.1%, which is more or less comparable to reports from Senegal (0.5%), Kenya (0.26%), Nigeria (1.5%) and Egypt (0.44%) [37,46,47]. However, higher prevalence of HCV-HBV-HIV triple co-infection was reported in Argentina (9.5%) and Iran (9.2%) [48,49]. For such variations, risk factors which accounts for HBV and HIV prevalence difference might work for the triple infection.

Despite absence of statistical significance difference in the mean levels of the liver enzymes between HIV-mono-infected and HIV-viral hepatitis co-infected individuals, raised ALT, AST and ALP were found in HIV-HBV, HIV-HCV and HIV-HBV-HCV co-infected individuals. However, in a study which was conducted in South Africa, 70% of HIV-HBV and HIV-HCV co-infected study participants had significantly elevated AST and ALT, 56% of them had elevated ALP [35]. Similarly, significantly raised ALT was found in 14% of HIV/HBV co-infections and 20% in HIV-HCV co-infected patients in India [50]. These liver enzyme levels difference between different studies may be due to difference in study design, duration of the viral hepatitis infection as well as the patient’s condition like having chronic alcoholism or other drug induced hepatotoxicity. In addition, HIV can also infect the hepatic or kupffer cells [51] that may farther contributes for the development of liver fibrosis and raised liver enzyme levels. However, the magnitude of the complication of the liver may be worse if the HIV positive patients co-infected with HBV and HCV as indicated in the above.

In the present study, there is no statistically significant CD4 count mean difference between HIV mono-infected, HIV-HBV and HIV- HCV co-infected study participants. However, study participants who had HIV-HBV co-infection in this study have the mean CD4 count (250 cells/mm^3^) which was incomparable with mean CD4 count of 141.6 cells/mm^3^ and 121 cells/mm^3^ in the studies which were conducted in South Africa and Nigeria respectively [35,46]. These controversial results may be due to the differences in the immune status of the individual who have been participated in the study or it may be due to the viral hepatitis. In individuals who have both HIV and HBV infections, there may be high HIV and HBV viral replication that may farther contribute for the impairment of the immune system of the patients.

In this study, the mean CD4 count (274 cells/mm^3^) was found in HIV-HCV co-infected study participants which is comparable with a mean CD4 count of 260 cells/mm^3^ and 288.6 cells/mm^3^ that were reported in Nigerian and Indian studies respectively [46,50]. Such high values of mean CD4 count in HIV-HCV co-infected study participants than study participants who had HIV-HBV co-infections were unclear. However, HIV-HCV co-infected study participants have relatively lower mean CD4 values than HIV mono-infected study participants. This low CD4 count in HIV-HCV co-infected may be associated with an increased HIV and HCV replication, reflecting the immunosuppressed state.

Among HIV-HBV-HCV infected individuals, the mean CD4 count was 125cells/mm^3^. This CD4 levels is comparable with the mean CD4 count of 116/mm^3^ which was reported in India [50]. Similarly in a study which was done in Nigeria, HIV-HBV-HCV infected individuals had the mean CD4 count of 106 cells/mm^3^[46]. However, all of these results showed the mean CD4 count of less than 200 cells/mm^3^. This could be due to the fact that the presence of both HBV and HCV in HIV positive individuals may highly contribute for the impairment of the immune system of an individual that may also further lead the person for the development of advanced HIV diseases. In addition, there is also inverse relationship between CD4 values and HIV diseases progression. However, the impact of viral hepatitis on the immune system and liver enzymes needs farther studies in both on HAART and HAART naïve HIV positive patients.

In the present study, there was also difference on the mean CD4 values in relation to gender. The mean CD4 value in the males was lower than females (249 cells/mm^3^ vs 299 cells/mm^3^). Similar findings were reported in studies which have been conducted in Nigeria [52] and Uganda [53]. Male study participants who had both HBV and HCV had also the lowest mean CD4 values. This lower CD4 count in males may be associated with their daily activities. In addition, males are mostly more muscular and may not be ready to accept their HIV, HBV and/or HCV results and they may develop mental stress that farther contribute for the impairment of their immune system or lower CD4 count. Farther more, males may spend most of their time with hard works for a long period of time and this may contribute for lower CD4 count. We have also analyzed the mean CD4 values in different age categories and the lowest mean CD4 values was observed in the age groups of 50 years and above. As age increases, there may be impairment of the immune system of the individuals specially HIV positive patients and older age groups may have severed HIV diseases progression than younger once.

In conclusion, the prevalence of viral hepatitis (HBV, HCV) among HIV positive individuals was higher than the prevalence of the respective viruses in the general population. Thus screening of HBV and HCV before initiation of antiretroviral treatment is mandatory for strict monitoring and a regular evaluation of liver enzyme levels and CD4 status in order to minimize the complication of the liver and for effective HIV treatment.

## Competing interests

The authors declare that they have no competing interests.

## Authors’ contributions

YW: Participated in conceived, designed and proposed the research idea, data collection, data entry, clearance, analysis and interpretation of the findings and drafting the manuscript and write up. MA: involved in data entry, clearance, analysis, and interpretation of the findings. FA: involved in data entry, clearance, analysis, and interpretation of the findings. YB: Participated in conceived, designed and proposed the research idea, data collection, data entry, clearance, analysis and interpretation of the findings. All authors involved in reviewing the manuscript and approval for publication.
